# Membrane Binding and Insertion of a pHLIP Peptide Studied by All-Atom Molecular Dynamics Simulations

**DOI:** 10.3390/ijms140714532

**Published:** 2013-07-12

**Authors:** Yonghua Deng, Zhenyu Qian, Yin Luo, Yun Zhang, Yuguang Mu, Guanghong Wei

**Affiliations:** 1 State Key Laboratory of Surface Physics, Key Laboratory for Computational Physical Sciences (Ministry of Education), and Department of Physics, Fudan University, 220 Handan Road, Shanghai 200433, China; E-Mails: 072019007@fudan.edu.cn (Y.D.); 09110190008@fudan.edu.cn (Z.Q.); 09110190006@fudan.edu.cn (Y.L.); 0529009@fudan.edu.cn (Y.Z.); 2 School of Biological Sciences, Nanyang Technological University, 60 Nanyang Drive, Singapore 637551, Singapore; E-Mail: ygmu@ntu.edu.sg

**Keywords:** pH low-insertion peptide (pHLIP), zwitterionic POPC, pH-triggered bilayer insertion, membrane binding, all-atom molecular dynamics simulations

## Abstract

Recent experiments in function mechanism study reported that a pH low-insertion peptide (pHLIP) can insert into a zwitterionic palmitoyloleoylphosphatidylcholine (POPC) lipid bilayer at acidic pH while binding to the bilayer surface at basic pH. However, the atomic details of the pH-dependent interaction of pHLIP with a POPC bilayer are not well understood. In this study, we investigate the detailed interactions of pHLIP with a POPC bilayer at acidic and basic pH conditions as those used in function mechanism study, using all-atom molecular dynamics (MD) simulations. Simulations have been performed by employing the initial configurations, where pHLIP is placed in aqueous solution, parallel to bilayer surface (system S), partially-inserted (system P), or fully-inserted (system F) in POPC bilayers. On the basis of multiple 200-ns MD simulations, we found (1) pHLIP in system S can spontaneously insert into a POPC bilayer at acidic pH, while binding to the membrane surface at basic pH; (2) pHLIP in system P can insert deep into a POPC bilayer at acidic pH, while it has a tendency to exit, and stays at bilayer surface at basic pH; (3) pHLIP in system F keeps in an α-helical structure at acidic pH while partially unfolding at basic pH. This study provides at atomic-level the pH-induced insertion of pHLIP into POPC bilayer.

## 1. Introduction

The pH low-insertion peptides (pHLIPs) have received significant attention in recent years due to their ability to target acidic tissues and selectively translocate polar, cell-impermeable molecules across cell membranes [1,2]. At pH values above seven, pHLIP in aqueous solution partitions to the surface of a lipid bilayer without inserting. Under acidic pH conditions, pHLIP inserts into a lipid bilayer and forms a transmembrane helix [1–5]. Due to its small size, pHLIP serves as a model peptide for studying membrane protein folding and bilayer insertion. Its folding energetics across a palmitoyloleoylphosphatidylcholine (POPC) lipid bilayer has been studied recently by fluorescence spectroscopy and isothermal titration calorimetry [4]. During the insertion process, pHLIP can translocate cargo molecules attached to its *C*-terminus via a disulfide, and release them in the cytoplasm of the cell [1,5,6]. Mutation study showed that Asp residues in the *C*-terminal region of pHLIP are critical for solubility and pH-dependent membrane insertion of the peptide [7,8]. Unlike other membrane active peptides [9], pHLIP helices cause minimal disturbance to phospholipid bilayers: Namely, they do not induce membrane leakage [1,6,10]. Owing to this exceptional property, many technologies have been developed for targeting, imaging, and drug molecule delivery using pHLIP [7,8,11–14].

A 35-aa peptide, with the sequence AEQNPIYWAR^10^YADWLFTTPL^20^LLLDLALLVD^30^ADEGT [3], is one of the pH low-insertion peptides. This pHLIP is a water-soluble peptide derived from the transmembrane helix C of the integral membrane proteins Bacteriorhodopsin [15] and displays a pH-triggered membrane insertion behavior [3]. In function mechanism study of the pH-dependent membrane insertion of pHLIP, the reported pH values for bilayer insertion and exit of this pHLIP peptide are 4.0 and 8.0, respectively [3]. In spite of well-performed experimental study, the atomic details of pH-induced bilayer insertion of this pHLIP peptide are not well understood.

Molecular dynamics (MD) simulations can provide detailed information on peptide-membrane interactions. A number of MD simulation studies have investigated the lipid interactions and the energetics of membrane insertion of different peptides/proteins [16–21]. The potential of mean force (PMF) for the transfer of pHLIP across lipid bilayers has been investigated by a coarse-grained MD study [16], which provides theoretical insights into the thermodynamics of pHLIP in membrane environment. However, the pH-dependent pHLIP-membrane interaction at atomic level is yet to be determined.

The goal of this study is to investigate the detailed interaction of pHLIP with a zwitterionic POPC lipid bilayer at different pH conditions. We have performed multiple, independent 200-ns MD simulations at acidic and basic pH starting from three different initial configurations where the initial structure of pHLIP is an α-helix. The reason of choosing the α-helical structure as the starting state of our MD simulations is discussed in detail in the Experimental Section. Noting, the pHLIP responds to the pH change from neutral to mildly acidic pH in biomedical experiments [7,8]. In the mechanism study of pHLIP in solution when POPC vesicles were employed instead of live cells [3], the used pHs were pH 4 and pH 8. Following this experimental study [3], such acidic and basic pHs (pH 4 and pH 8) were chosen for our simulation study [3]. In the initial configurations, pHLIP is placed parallel to bilayer surface (system S), partially-inserted (system P), or fully-inserted (system F) in the POPC bilayer. The membrane binding process and insertion behavior of pHLIP, as well as the pHLIP-POPC interactions at acidic and basic pH are to be investigated. This study provides molecular insight into the mechanism of pH-triggered membrane insertion for pHLIP peptides.

## 2. Results and Discussion

To mimic the experimental acidic pH (pH 4.0) condition [3], the side chains of Asp, Glu, Arg, and the *N*-terminus are protonated (Asp0, Glu0, Arg^+^, NH_3_^+^). In order to examine the role of protonation of Asp/Glu residues in the transmembrane and *C*-terminal region on the bilayer insertion of pHLIP, and exclude the influence of *C*-terminus it is taken as deprotonated (COO-) (with the same protonation state as that at basic pH), while COO- and COOH coexist around pH 4.0. In a basic pH (pH 8.0) environment, the side chains of Asp and Glu and the *N*- and *C*-termini are deprotonated (Asp-, Glu-, NH_2_ and COO-), and the side chain of Arg is protonated (Arg^+^). The pHLIP carries one net positive charge at this acidic pH and six net negative charges at this basic pH. It should be noted that, in reality, the pK of each Asp/Glu shifts with the surrounding environment (the protonation state of Asp/Glu will change accordingly). As the calculation of pK is very computational costly, we neglect the pK shift in our MD simulations, as done recently by other groups [18,19,22–24].

**Figure 1:**
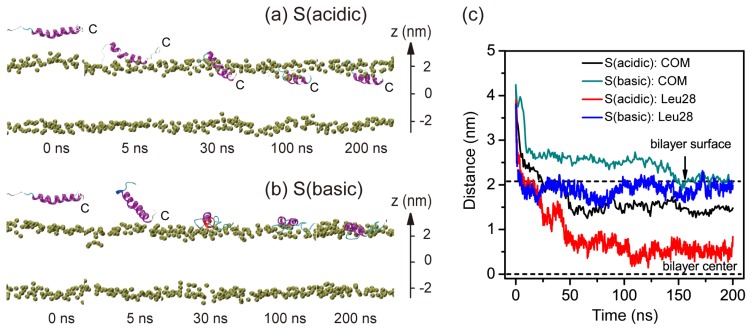
Detailed analysis of the two MD trajectories for system S at acidic and basic pHs. Snapshots at five different time points in the MD runs of (**a**) S (acidic) and (**b**) S (basic). The peptide is shown in ribbon. The phosphorous atoms of the lipids are shown in tan spheres to display the bilayer surface. The other atoms of POPC lipid molecules and water molecules are not shown for clarity. The bilayer center is set at *z* = 0; (**c**) The *z*-position of the center-of-mass (COM) of pHLIP backbone atoms and the z-position of the most-deeply embedded atom in residue Leu28 as a function of time. The two dashed black lines at *z* = 2.05 nm and *z* = 0 nm correspond to the bilayer surface (the headgroup phosphorus atoms) of the upper leaflet and the bilayer center, respectively.

We have carried out multiple, independent 200-ns MD simulations starting from the three different initial states S, P, F described in Experimental Section. Spontaneous bilayer insertion of pHLIP at acidic pH is relatively rare in our performed MD simulations at 315 K. We have launched five MD runs for system S (acidic) (data not shown), while insertion event is only observed in one MD trajectory. MD runs of the P and F systems are used to further confirm the insertion property observed in S (acidic). Here we present only the results of six representative MD runs, named as S (acidic), S (basic), P (acidic), P (basic), F (acidic), and F (basic). We first investigate the pH-dependent insertion behavior of pHLIP in system S, where pHLIP was initially placed in aqueous solution with its backbone parallel to the POPC bilayer surface and the center-of-mass (COM) of the pHLIP backbone is 2.2 nm away from the membrane surface (see the snapshot at *t* = 0 ns in Figure 1a,b).

### 2.1. In Aqueous Solution, pHLIP Can Spontaneously Insert into POPC Bilayer at Acidic pH, while Binds to the Membrane Surface without Inserting at Basic pH

Figure 1 presents the pH-dependent pHLIP-POPC interaction in the two MD trajectories of S (acidic) and S (basic). The snapshots generated at five different time points in the two MD runs are displayed in Figure 1a,b. The time evolution of the insertion depth of pHLIP in POPC lipid bilayer is shown in Figure 1c. The bilayer insertion depth is probed by two parameters: One is the z-position of the COM of peptide backbone and the other the *z*-position of the most deeply inserted atom of residue Leu28. In acidic pH environment, the pHLIP moves toward the hydrophilic headgroup of the POPC bilayer and is adsorbed to bilayer surface within the first 5 ns, with its *C*-terminal residues binding to the headgroup region of the bilayer. This is followed by a spontaneous insertion of the *C*-terminal residues into the hydrophobic tail region of the bilayer and at *t* = 25 ns, the *C*-terminal residue Leu28 is located 0.5 nm below the phosphorous atoms (*z* = 2.05 nm) (see red curve in Figure 1c). It takes some time for the peptide to across the headgroup region. A full insertion of the pHLIP inside POPC bilayer is observed at *t* = 50 ns, with a z-position of 1.5 nm for backbone COM and 0.55 nm for the most deeply inserted atom of Leu28. Thereafter the peptide continues to move toward the hydrophobic center and at *t* = 100 ns, the *z*-position of the deepest atom of Leu28 reaches to 0.5 nm, although the backbone COM is still located at *z* = 1.5 nm. Interestingly, although the pHLIP is initially placed parallel to the membrane surface, the *C*-terminal residues firstly binds to the bilayer surface and directs the whole chain to insert into the bilayer. This is in accordance with the experiment results that the pHLIP insertion across a membrane is unidirectional with the *C*-terminal going first to insert [1,7].

Similar MD simulations studied on other peptides such as alamethicin [25], magainin [26], protegrin-1 [27], Buforin II [22,24], and WALP [23] have been reported to investigate peptide-lipid interactions. Insertion of WALP peptides (ace-AWW-(LA)_5_-WWA-ame and ace-GWW-(LA)_8_L-WWA-ame) into DPPC bilayer was observed in high-temperature (>80 °C) MD simulations [23]. Interestingly, spontaneous insertion of Buforin II and Transportan 10 peptides into POPC bilayer was also observed in recent MD simulations at 300 K [19,22,24].

At basic pH, the peptide is also quickly adsorbed to the bilayer surface within the first 5 ns (see Figure 1b), while its backbone COM (*z* = 2.7 nm) is still in the aqueous solution (see Figure 1c). At *t* = 30 ns the peptide stays at the water-bilayer interface with its backbone parallel to bilayer surface (see the snapshot in Figure 1b) and a z-position of backbone COM of 2.7 nm. At *t* = 150 ns, the backbone COM and the most deeply inserted atom of Leu28 both reach to *z* = 2.05 nm (the location of phosphorous atom in the POPC headgroup). The peptide remains there during the remainder of the 200-ns MD simulations. This finding is in good agreement with fluorescence experiments showing that the pHLIP binds to the surface of POPC lipid bilayers at pH 8.0 [3].

To examine the conformational change during the bilayer binding and insertion process of pHLIP, we have plotted in Figure S1 the DSSP profile of pHLIP. At acidic pH, the helix unfolds from the water-exposed *N*-terminal region, during the process of the *C*-terminal residues of pHLIP across the headgroup region. After the peptide fully inserts into the bilayer (after 60 ns), some of the unfolded *N*-terminal residues (residues 14–19) start to refold and a helix spanning residue 13–27 is observed at 125 ns. This helical structure maintains during the remainder of the 200-ns MD simulation. At basic pH, the helix unfolds from the *C*-terminal region (residues 24–28) during the bilayer binding process, which is likely due to the strong electrostatic interactions of the negatively charged D24, D30, D32, and E33 residues with the positively charged choline group in the headgroup region of POPC. However, refolding event is not observed within the 200-ns MD trajectory.

### 2.2. When Partially Pre-Inserted in POPC Bilayer, pHLIP Can Insert Deep into the Bilayer at Acidic pH while Moves towards the Bilayer Surface at Basic pH

**Figure 2:**
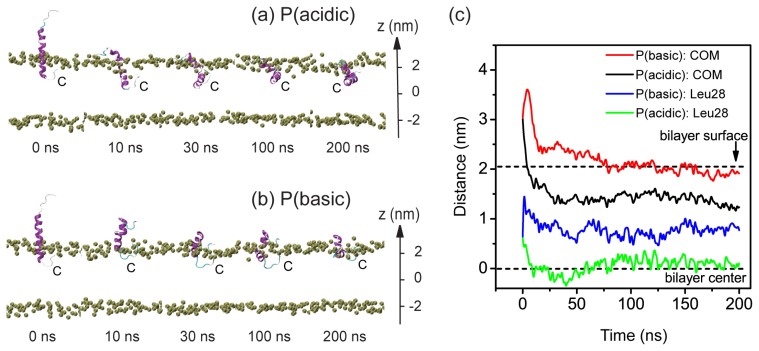
Detailed analysis of the two MD trajectories for system P at acidic and basic pHs. Snapshots at five different time points in the MD runs of (**a**) P (acidic) and (**b**) P(basic). The peptide is shown in ribbon. The phosphorous atoms of the lipids are shown in tan spheres to display the bilayer surface. The other atoms of POPC lipid molecules and water molecules are not shown for clarity. The bilayer center is set at *z* = 0; (**c**) The *z*-position of the center-of-mass (COM) of pHLIP backbone atoms and the *z*-position of the most deeply inserted atom in residue Leu28 as a function of time. The two dashed black lines at *z* = 2.05 nm and *z* = 0 nm corresponds to the bilayer surface (the headgroup phosphorus atoms) of the upper leaflet and the bilayer center, respectively.

To further examine the pH-dependent bilayer insertion properties of pHLIP, we have launched two 200-ns MD runs for the partial-insertion system (system P) at acidic and basic pH solution conditions. The two MD runs are labeled as P (acidic) and P (basic). Figure 2 shows the snapshots taken at five different time points and the time evolution of bilayer insertion depth of pHLIP. The bilayer insertion depth is described by two parameters: one is the *z*-position of the peptide backbone COM and the other the *z*-position of the most deeply inserted atom of residue Leu28. In the initial state (*t* = 0 ns), the z-position is 3.0 nm for peptide COM and 1.5 nm for Leu28 (see Figure 2c). It can be seen from Figure 2a,c) that at acidic pH condition, the pHLIP moves towards the bilayer center. The *z*-positions of peptide COM and residue Leu28 both decrease with simulation time, reaching to respectively *z* = 1.5 nm and 0.2 nm at *t* = 100 ns. These two parameters remain respectively around the two values in the left period of MD runs and the peptide stays inside the bilayer. This insertion depth is very similar to the one observed in the MD run of S (acidic). In contrast, at basic pH the pre-inserted *C*-terminal residues move towards the hydrophilic headgroup region of the upper leaflet and have the tendency to exit from the hydrophobic region of bilayer (see Figure 2b,c). The *z*-position of residue Leu28 is 1.0 nm at *t* = 200 ns, while it is 0.6 nm at *t* = 0 ns, indicative of a tendency of moving to the bilayer surface. The peptide COM is located at *z* = 2.0 nm (the *z*-position of bilayer surface) during the last 50 ns. Interestingly, although we place the pHLIP perpendicular to the plane of POPC bilayer in the initial state, the peptide appears to move towards an in-pane orientation and stays near the surface of POPC bilayer. These results provide further supports on the finding observed in the two MD runs of S (acidic) and S (basic) given in Figure 1: pHLIP have a tendency to insert into the bilayer at acidic pH while binds to the bilayer surface at basic pH. It is noted that the surface-bound pHLIP is in a partially folded state.

The conformational change of pHLIP during the membrane entry and exit process is also probed by plotting the DSSP profile in Figure S2. At acidic pH, most of the helical region remains with a kink at residues W14 and L15 during the bilayer insertion process. In contrast, at basic pH condition, the helix unfolds partially during the bilayer exit process. In the final conformation, the initial helix spanning residue 14–18 is lost, which is likely due to the strong electrostatic interactions of residue D13 and D24 with POPC headgroups (see below for the electrostatic interaction energy given in Figure 6). The conformational deformation of pHLIP helix observed here and that in S (basic) system is probably correlated with the strong electrostatic interactions with lipid headgroups. Interestingly, in a recent MD study on membrane entry of buforin II peptide, it was reported that the extent of structural deformation appeared directly related to the formation of increased lipid interactions [24].

**Figure 3:**
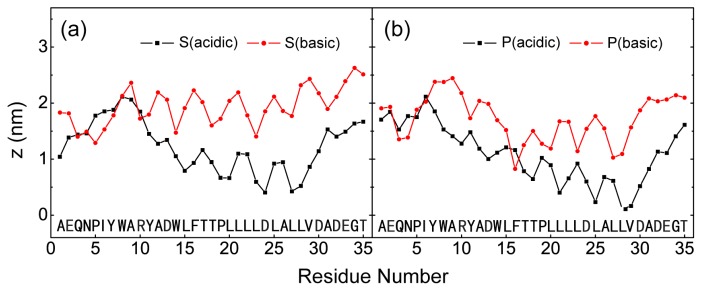
The *z*-position of the most deeply inserted peptide atom of each residue in POPC bilayer. The results are from four different MD runs: (**a**) S (acidic) and S (basic); (**b**) P (acidic) and P (basic). For each MD run, the *z*-position is an average of the last 50 ns.

In order to compare the bilayer insertion depth of pHLIP in the four different MD runs, starting from S and P configurations, we plot the z-position of the most deeply inserted atom of each residue in Figure 3. It can be seen from Figure 3a,b that starting from two states (S and P) with different starting positions and orientations of the peptides relative to the bilayer surface, MD runs S (acidic) and P (acidic) at acidic pH both lead to a bilayer-insertion state of the peptide, while MD runs S (basic) and P (basic) at basic pH result in a surface-bound state of the pHLIP peptide. Interestingly, we observe that the most hydrophobic region spanning residues W14-V29 are buried deep in the POPC bilayer, with residue L28 being the most deeply bilayer-inserted residue. This is also the reason that we use residue L28 to monitor the insertion depth of the peptide in Figures 1 and 3.

### 2.3. When Fully Pre-Inserted inside POPC Bilayer, pHLIP Keeps Intact α-Helical Structure at Acidic pH while the Helix Unfolds at Basic pH

**Figure 4:**
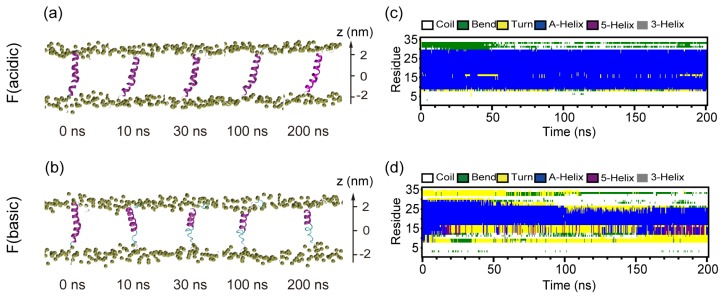
Detailed analysis of the two MD trajectories (F (acidic) and F (basic)) for full-insertion system at acidic and basic pHs. Snapshots at five different time points in the MD runs of (**a**) F (acidic) and (**b**) F (basic). The peptide is shown in ribbon. The phosphorous atoms of the lipids are shown in tan spheres to display the bilayer surface. The other atoms of POPC lipid molecules and water molecules are not shown for clarity. The bilayer center is set at *z* = 0. The secondary structure profiles of the pHLIP in the MD runs of (**c**) F (acidic) and (**d**) F (basic).

In order to probe the stability and the conformation change of the pHLIP inside the POPC bilayer, we have performed MD simulations with pHLIP fully-inserted in the bilayer at both acidic and basic pH. Figure 4 shows the snapshots taken at five different time points and the time evolution of secondary structure profile of the pHLIP. The initial state of pHLIP in the two MD runs is a trans-bilayer α-helix. During the full period of 200-ns MD runs of F (acidic), the initial α-helix keeps intact. However, it unfolds in the MD run of F (basic) and loses 30% of its initial helical structure at *t* = 10 ns. Helix unfolding mainly initiates from the *C*-terminus. With the increase of simulation time, more helical structure is lost and only 40% of initial α-helix remains at *t* = 200 ns. It is expected that the α-helix will unfold completely if the simulation time is long enough. The helix unfolding implies that α-helical structure is not favorable for membrane-bound pHLIP at basic pH. This is consistent with the experimental results that the pHLIP in the membrane unfolds, from helix to coil, before the exit when triggered by acidity change of pH from 4 to 8 [3]. However, the spontaneous exit was not observed in our 200 ns MD run, likely due to the 200 ns being too short to observe the spontaneous exit of pHLIP (the estimated exit time is 65 milliseconds [3]).

### 2.4. The Protonation of Transmembrane Residues Asp and the C-Terminal Residues Asp/Glu Facilitates Their Bilayer Entry Due to the Increased Hydrophobicity of pHLIP at Acidic pH, while the Strong Electrostatic Interaction between the Deprotonated Asp/Glu Residues and Lipid Headgroups Hinders the Bilayer Insertion at Basic pH

**Figure 5:**
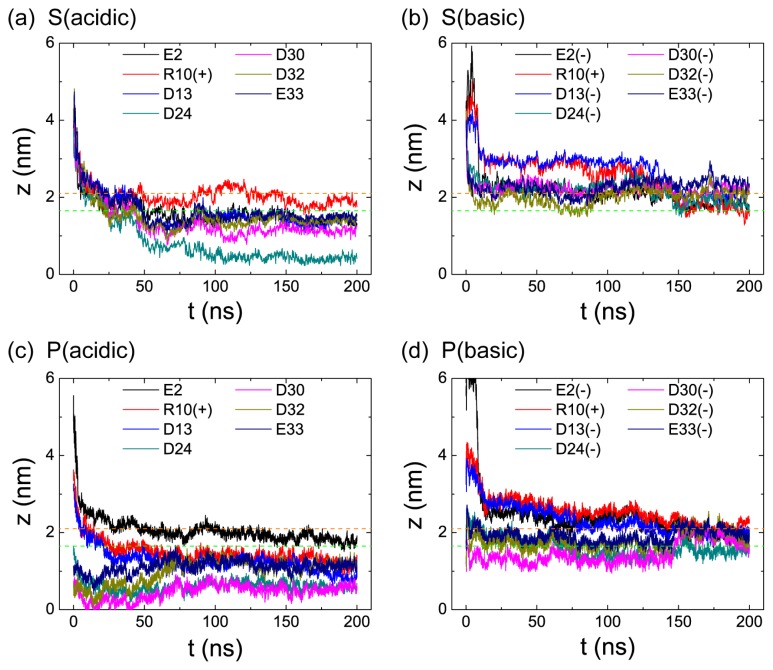
Time evolution of the minimum distance from bilayer center (*z* = 0) for each of the seven charged residues (E2, R10, D13, D24, D30, D32, and E33) in the four MD runs of S (acidic), S (basic), P (acidic), and P (basic).

To identify the critical residues and the important physical interactions for pH-dependent pHLIP-POPC interaction, we have first calculated the time evolution of the minimum distance from bilayer center (*z* = 0) for each of the seven polar residues (E2, R10, D13, D24, D30, D32, and E33) in the four MD runs of S (acidic), S (basic), P (acidic), and P (basic). The results are given in Figure 5. It can be seen from Figure 5 that for S system, at acidic pH (Figure 5a), pHLIP binds to POPC bilayer surface within the first 25 ns. The transmembrane residue D24 penetrates into the lipid tail region at ~30 ns. Bilayer insertion of the *C*-terminal residues D30 and D32 is observed at *t* = 50 ns. This is followed by the membrane entry of residues D13, E33, and E2. During the last 50 ns, all of the Asp and Glu residues stay in the hydrophobic tail region of POPC bilayer, while the positively charge R10 still binds to the hydrophilic headgroup region, which is likely due to strong attractive electrostatic interaction between the positively charged R10 and the negatively charged phosphate group in the headgroups. However, at basic pH (Figure 5b), all of the Asp and Glu residues bind to the headgroup region of POPC bilayer, which is likely due to the strong attractive electrostatic interaction between the negatively charged E2, D13, D24, D30, and D32 and the positively charged choline groups. When pHLIP partially pre-inserted in the POPC bilayer (P system), at acidic pH (Figure 5c), transmembrane residue D24 and the *C*-terminal residue D30 insert deep into POPC bilayer, resulting in membrane entry of R10 and D13. During the last 50 ns, all of the Asp and Glu residues are located in the hydrophobic tail region of POPC bilayer, with residue D24 and D30 being buried most deeply. However, at basic pH (Figure 5d), residues D30(-) and D32(-) move to the lipid hydriphilic headgroup region from the hydrophobic tail region. During the last 50 ns, all of the Asp and Glu residues bind to the headgroup region of POPC bilayer.

**Figure 6:**
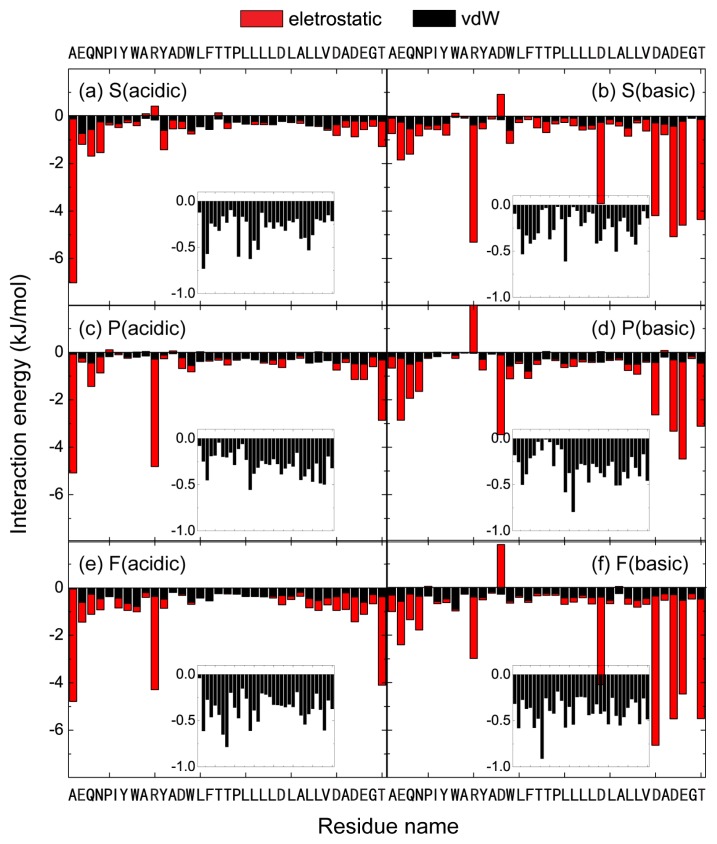
The time-averaged interaction energy between each individual residue of pHLIP and POPC lipids (per lipid) in the six different MD runs: (**a**) S (acidic); (**b**) S (basic); (**c**) P(acidic); (**d**) P(basic); (**e**) F(acidic); (**f**) F(basic). The interaction energy is averaged over the last 50 ns of each MD run. The residue-based interaction energy is decomposed into the electrostatic and van der Waals (*vdW*) terms.

We then plot in Figure 6 the interaction energy between each amino acid residue and POPC lipid bilayer (per lipid) for the six different MD runs starting from the three different initial states. For each MD run, the interaction energy is averaged over the last 50 ns and decomposed into electrostatic and van der Waals (*vdW*) components. To compare the *vdW* interaction of each residue with POPG lipids at acidic and basic pH conditions, we present in the inset the *vdW* interaction energy using a different scale. It appears that the insertion depth is not directly correlated with the total *vdW* interaction energy between each residue and lipid molecule (tail and headgroup). Similar *vdW* interaction energies are seen in the six different systems. It can be seen from Figure 6 that the interaction energies between charged residues and POPC lipid are much stronger with respect to uncharged residues, indicating that electrostatic interactions play important role on the pH-dependent pHLIP-POPC interaction. The importance of electrostatic interactions between charged residues and lipid headgroups on the interaction of amyloid peptides with zwitterionic/charged lipid bilayers has also been reported in recent MD studies [28–31]. By comparing the peptide-lipid interaction energy in the left three panels with those in the right three panels in Figure 6, we see that the *C*-terminal polar residues Asp30, Asp32, and Glu33 display much stronger electrostatic interactions with POPC lipids at basic pH, which may prevent pHLIP from inserting into the lipid bilayer. The pHLIP has a net charge of −6 at basic pH and +1 at acidic pH. The peptide at basic pH is more hydrophilic, energetically more favorable to bind to hydrophilic headgroups of POPC bilayer, whereas the pHLIP at acidic pH is more hydrophobic, energetically more favorable to stay inside the hydrophobic tail region of POPC bilayer. This view is supported by the time-average potential energy calculation for the whole system (consisting of pHLIP, lipid bilayer, water molecules, and counterions) over the last 50 ns. Given the fact that the simulation timescales are too short to estimate the entropic contributions accurately, our analysis is only qualitative and indicative. The total potential energies of S (acidic), P (acidic), and F (acidic) system are −5590, −5818, and −5835 kJ/mol, respectively, with the potential energy of F (acidic) system being the lowest (−5835 kJ/mol). The total potential energies of S (basic), P (basic), and F (basic) are −8784, −9166, and −8475 kJ/mol, respectively, with the potential energy of F (basic) system being the highest (−8475 kJ/mol).

**Figure 7:**
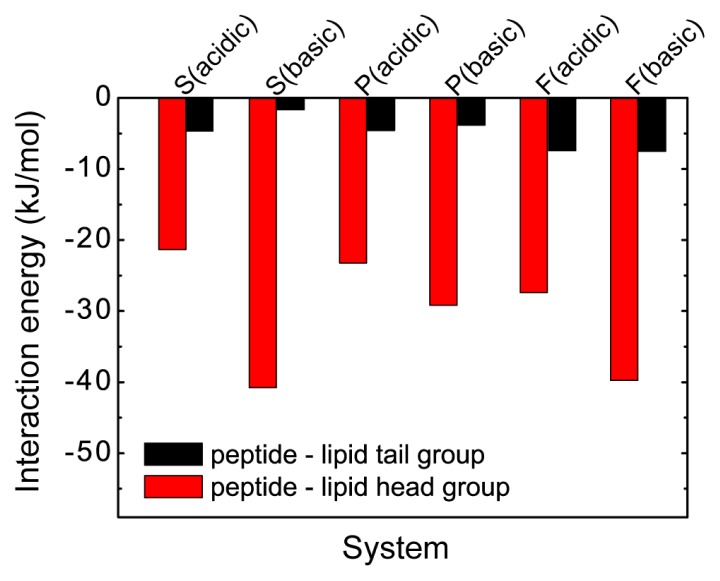
The interaction energy of pHLIP with POPC lipid head group (red) and tail group (black) (per lipid) in the six different MD runs: S (acidic), S (basic), P (acidic), P (basic), F (acidic), and F (basic). The data are averaged over the last 50 ns for each MD run.

To understand further the physical driving forces underlying the pH-dependent pHLIP-POPC lipid interaction, we have calculated the interaction energies of pHLIP with the polar headgroup and the hydrophobic tail group of POPC lipid (per lipid) in the six different MD runs. The results are given in Figure 7, showing that the interaction energy of the peptide-lipid headgroup is much larger than that of peptide-lipid tail group in both acidic and basic pH conditions, indicating that pHLIP peptide has a strong interaction with the lipid head group. Moreover, the peptide-lipid head group interaction in basic pH solution condition is much stronger than that in acidic pH condition, which may induce the peptide to bind to POPC bilayer surface at basic pH rather than to insert into the bilayer. The results in Figure 6, together with those in Figures 5 and 7, indicate that at acidic pH, the protonation of the transmembrane residues Asp and the *C*-terminal residues Asp/Glu results in an increased hydrophobicity of pHLIP, facilitating their bilayer entry, while at basic pH, the strong electrostatic interaction between Asp/Glu residues and lipid headgroups hinders bilayer insertion of the pHLIP peptide. Notably, it was suggested in a recent MD simulation study that the electrostatic attraction between transportan 10 and POPC headgroups is the main bottleneck for the peptide across the bilayer [19].

### 2.5. Memebrane Insertion of pHLIP Does Not Disrupt the Integrity of POPC Bilayer Structure

**Figure 8:**
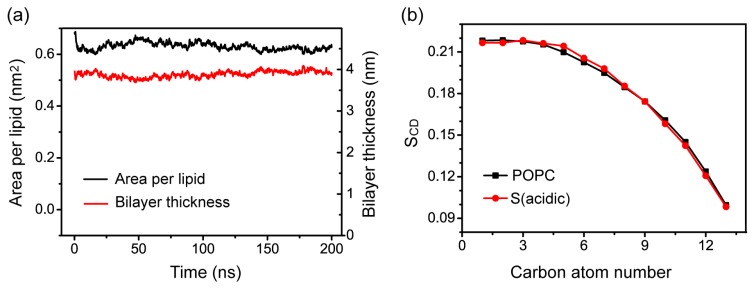
Characterization of the ordering of POPC bilayer. (**a**) Time evolution of area per lipid and bilayer thickness in simulation S (acidic); (**b**) Time-averaged order parameter *S*_CD_ of sn-1 chain of POPC lipids over the last 50-ns of MD run of S (acidic). For comparison, the *S*_CD_ of sn-1 chain obtained from a 20 ns MD run for pure POPC lipid bilayer is also presented.

Our S (acidic) MD simulation shows that pHLIP can spontaneously insert into POPC bilayer at acidic pH (see Figure 1a). Recent experimental studies reported that membrane insertion of pHLIP causes minimal disturbance to POPC bilayers and does not induce membrane leakage [1,3,6,10]. To examine whether bilayer insertion of pHLIP at acidic pH would perturb the structure of POPC bialyer, we probe the area per lipid and the POPC bilayer thickness as a function of simulation time in the MD run of S (acidic). Figure 8a shows that the values of area per lipid, and bilayer thickness, remain almost constant during the last 50 ns of MD simulation. Our calculated area per lipid and bilayer thickness is 0.63 ± 0.02 nm^2^ and 3.88 ± 0.02 nm, respectively, consistent with the experimentally measured values of 0.68 nm^2^ for area per lipid and 3.76 nm for phosphorus-phosphorus distance [32]. We also examine the influence of bilayer-insertion of the pHLIP on the ordering of lipid tail by calculating lipid tail order parameter *S*^CD^. Figure 8b presents the average *S*_CD_ value of acyl chain 1 (sn-1) of POPC lipids over the last 50-ns MD run. For comparison, *S*_CD_ of pure POPC lipid bilayer from a 100-ns MD simulation is also given in Figure 8b. It can be seen from Figure 8b that the *S*_CD_ values of POPC from MD run of S (acidic) overlaps well with that of the pure POPC lipid bilayer. The values of *S*_CD_, together with the calculated bilayer thickness and area per lipid, demonstrate that membrane insertion of pHLIP has negligible perturbation on the structure of POPC bilayer.

We have also examined the membrane perturbation of pHLIP in system F, where the helix spans the bilayer by calculating the area per lipid, bilayer thickness, and lipid tail order parameter *S*_CD_ of sn-1 chain. Figure S3a shows that the values of area per lipid and bilayer thickness remain almost constant during the last 50 ns of MD simulations, in good agreement with the results given in Figure 8a for S (acidic) system. It can be seen from Figure S3b that, although the greatest extent of bilayer perturbation is observed in F (acidic) system where the pHLIP spans the POPC bilayer most extensively, the *S*_CD_ value in F (acidic) system is still within the error bar of pure POPC systems, indicating that insertion of pHLIP across a POPC bilayer does not disrupt the integrity of the membrane structure.

## 3. Experimental Section

### 3.1. pHLIP-POPC System

The amino acid sequence of pHLIP used in this study is AEQNPIYWAR10 YADWLFTTPL20 LLLDLALLVD30 ADEGT. It has been reported that pHLIP is unstructured in solution and displays α-helical structure when binding to membrane surface or inserting inside bilayer at pH 4.0 [3]. As the coil-helix transition time for this peptide at water-bilayer interface is about 1 s [3], it is out of reach to capture the conformational transition from random coil to α-helix using MD simulations at physiological temperatures. Moreover, recent experimental study reported that at pH 4.0 the pHLIP forms a helix on the POPC bilayer surface, followed by bilayer insertion [3]. As a first step to understand the pH-triggered bilayer insertion of pHLIP by conventional MD simulations, we selected the preformed helical structure as a starting state for our MD simulations. This strategy has been used by us, and other groups, for the study of bilayer insertion and membrane perturbation of antimicrobial/amyloidogenic/cell-penetrating peptides [18–20,22,24,28,33]. This helical structure is taken from the 73–107 region of transmembrane helix C in the NMR structure of protein Bacteriorhodopsin (pdb ID: 1R2N) [15]. The core of the pHLIP structure is an α-helix running from residues 8–29 (corresponding to the region from residue W80 to V101 in 1R2N).

The lipid bilayer, consists of 2 × 64 POPC molecules (*i.e*., 64 lipids in each leaflet) and the initial coordinates are obtained from a previous computational study of a pure POPC membrane [34]. The pHLIP was initially placed in three different positions with respect to the bilayer. These initial configurations are denoted as: (I) system S, in which pHLIP was initially placed in aqueous solution with its backbone parallel to the POPC bilayer surface and the center-of-mass (COM) of the pHLIP backbone is 1.8 nm away from the membrane surface (see the snapshot at *t* = 0 ns in Figure 1a,b); (II) system P, where pHLIP was partially-inserted in POPC bilayer with the *C*-terminal residues 24–35 embedded inside lipid bilayer (see the snapshot at *t* = 0 ns in Figure 2a,b); (III) system F, where pHLIP was fully-inserted in POPC bilayer with the pHLIP helix spanning the bilayer (see the snapshot at *t* = 0 ns in Figure 4a,b). All pHLIP-bilayer systems are fully solvated in water. Counterions (Cl^−^ or Na^+^) are added to neutralize the system. The VMD software was used to display the systems [35].

### 3.2. Molecular Dynamics Simulations

All MD simulations have been performed in the isothermal-isobaric (NPT) ensemble using the GROMACS 3.3 software package [36]. The Gromos 87 force field [37] is used to describe the peptide and bilayer interactions. A water molecule is modeled by the simple point charge (SPC) model [38]. Bond length of the peptides and lipids are constrained with LINCS [39] and water geometries are constrained with SETTLE [40]. This allows an integration time of 2 fs. Long-range electrostatic interactions are calculated using the Particle Mesh Ewald (PME) [41] method, as recommended for membrane simulations [42,43]. Van der Waals (*vdW*) interactions are calculated using a cutoff of 1.4 nm [42]. The lipid bilayer, water, peptide, and ions are weakly coupled (with a coupling constant of 0.1 ps) to temperature bath [44] separately at *T* = 315 K, above the gel-liquid crystal phase transition temperature (270 K) of POPC lipid bilayer [45]. The pressure is also weakly coupled (with a coupling constant of 1.0 ps, and compressibility of 4.5 × 105 bar) to 1 bar using semi-isotropic coupling scheme, in which the lateral (parallel to bilayer surface) and perpendicular (along bilayer normal) pressure are coupled independently [42]. A 200-ns MD simulation has been carried out for each system (S, P, F system) in both acidic and basic pH environment and there are six MD runs in total. The six MD runs are labeled as S (acidic), S (basic), P (acidic), P (basic), F (acidic), and F (basic). Here the letters S, P, F represent surface, partial-insertion, and full-insertion system, respectively. All MD simulations have been performed using periodic boundary condition in a rectangular box. A summary of the MD setup details is given in Table 1.

### 3.3. Analysis

All of the analyses have been done using our in-house developed codes and the analysis tools available in the GROMACS 3.3 package [36]. The ordering of POPC lipid bilayer is characterized by lipid tail order parameter (S_CD_), bilayer thickness, and the area per lipid. The bilayer thickness is estimated by the average of the phosphorus-phosphorus distance. The Lipid tail order parameter S_CD_ is calculated using the formula: *S*_CD_ = 0.5 × 〈3cos^2^θ − 1〉, where, θ is the angle between the C-H bond vector (in the simulation) or the C-D bond vector (in the experiment) and the bilayer normal. The angular brackets imply an average over time and lipid molecules [46,47]. The secondary structure analysis is performed using DSSP program [48]. The insertion depth of pHLIP peptide in POPC lipid bilayer is estimated by the *z*-position of the COM of peptide backbone and the *z*-position of the most deeply inserted atom of residue Leu28 in the bilayer. The *z*-position is averaged over the last 50 ns for each MD run. The interaction energy of each amino acid residue with the POPG lipid bilayer (per lipid), and the interaction energy of pHLIP with POPC lipid head group and tail group (per lipid) in the six different MD runs are also calculated, averaged over the last 50 ns of each MD run.

## 4. Conclusions

In summary, we have investigated the detailed interactions of pHLIP with POPC bilayers at acidic and basic pH conditions by performing six 200-ns all-atom MD simulations starting from three different configurations. When the peptide was placed in aqueous solution parallel to the bilayer surface, pHLIP is quickly adsorbed to the bilayer surface and can spontaneously insert in POPC bilayer at acidic pH, while it binds to the membrane surface without inserting at basic pH. Calculation of lipid order parameters demonstrates that bilayer insertion of pHLIP does not disrupt the structure of POPC bilayer, consistent with recent experimental studies showing that membrane insertion of pHLIP causes minimal disturbance to POPC bilayers and does not induce membrane leakage [1,6,10]. When the peptide was partially inserted in POPC bilayer, it can insert deep into the bilayer at acidic pH while moves towards the bilayer surface at basic pH. When it was fully inserted inside POPC bilayer, pHLIP keeps intact α-helical structure at acidic pH while the helix unfolds at basic pH. The pHLIP at acidic pH is more hydrophobic, energetically more favorable to stay inside the hydrophobic tail region of POPC bilayer At acidic pH, the protonation of transmembrane residues Asp and the *C*-terminal residues Asp/Glu facilitates their bilayer insertion due to the increased hydrophobicity of pHLIP, while at basic pH, the strong electrostatic interaction between Asp/Glu residues and lipid headgroups hinders the bilayer insertion. The total potential energy analysis indicates that it is energetically more favorable for the pHLIP to stay inside the membrane as an α-helical structure at acidic pH while bind to the membrane surface at basic pH. This study provides atomic details for the pH-dependent pHLIP-POPC interaction and improves our understanding toward the molecular mechanism underlying the pH-triggered bilayer insertion of pHLIP.

## Supplementary Information



## Figures and Tables

**Table 1:** Summary for the setup details of the six 200-ns MD runs at 315 K for three different systems S, P, and F. For each system, we describe the name of the system, the initial membrane insertion state of pHLIP, the name of MD runs, the initial state of each MD run, the total number of atoms, the size of simulation box and the length of each MD run.

System	Insertion state of pHLIP	Name of MD run	Initial state	Number of atoms	Simulation box size (nm^3^)	Length of MD run (ns)
S	Surface	S (acidic)	Figure 1a	27,083	6.6 × 6.5 × 9.0	200
	Surface	S (basic)	Figure 1b	27,066	6.6 × 6.5 × 9.0	200

P	Partial insertion	P (acidic)	Figure 2a	24,347	6.5 × 6.5 × 9.0	200
	Partial insertion	P (basic)	Figure 2b	24,345	6.5 × 6.5 × 9.0	200

F	Full insertion	F (acidic)	Figure 4a	16,808	6.4 × 6.3 × 6.5	200
	Full insertion	F (basic)	Figure 4b	16,789	6.4 × 6.3 × 6.5	200
