# Supplementary material for: Membrane Binding and Insertion of a pHLIP Peptide Studied by All-Atom Molecular Dynamics Simulations
Source: Int J Mol Sci. 2013 Jul 12;14(7):14532–49. doi: 10.3390/ijms140714532 (PMC3742258; doi:10.3390/ijms140714532)

Supplementary Information

Figure S1. The secondary structure profile of the pHLIP in the MD runs of (**a**) S (acidic) and (**b**) S (basic).

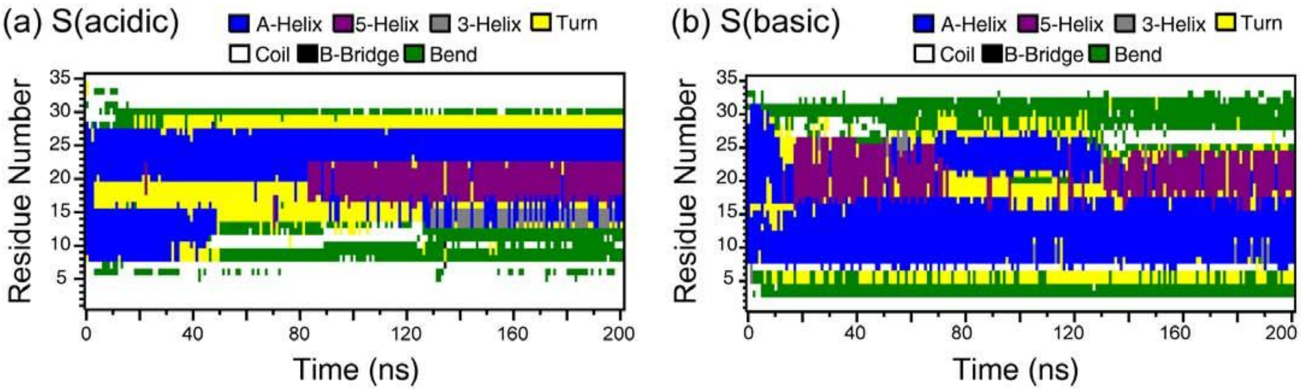

Figure S2. The secondary structure profile of the pHLIP in the MD runs of (**a**) P (acidic) and (**b**) P(basic).

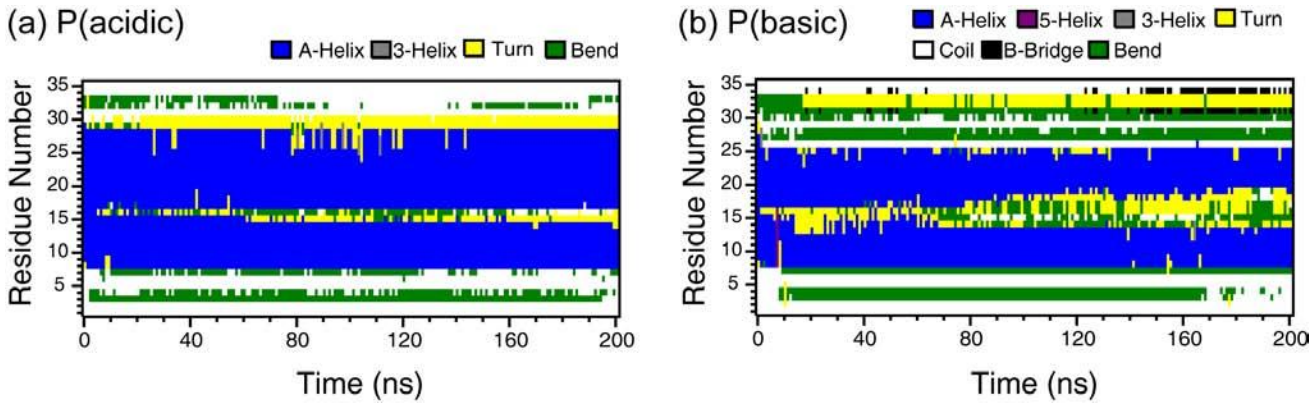

Figure S3. Characterization of the ordering of POPC bilayer in F system. (**a**) Time evolution of area per lipid and bilayer thickness in simulation F (acidic) (upper panel) and F (basic) (lower panel); (**b**) Time-averaged order parameter S_{CD} of sn-1 chain of POPC lipids over the last 50-ns of MD runs P (acidic) and P (basic). For comparison, the S_{CD} of sn-1 chain obtained from a 20 ns MD run for pure POPC lipid bilayer is also presented.

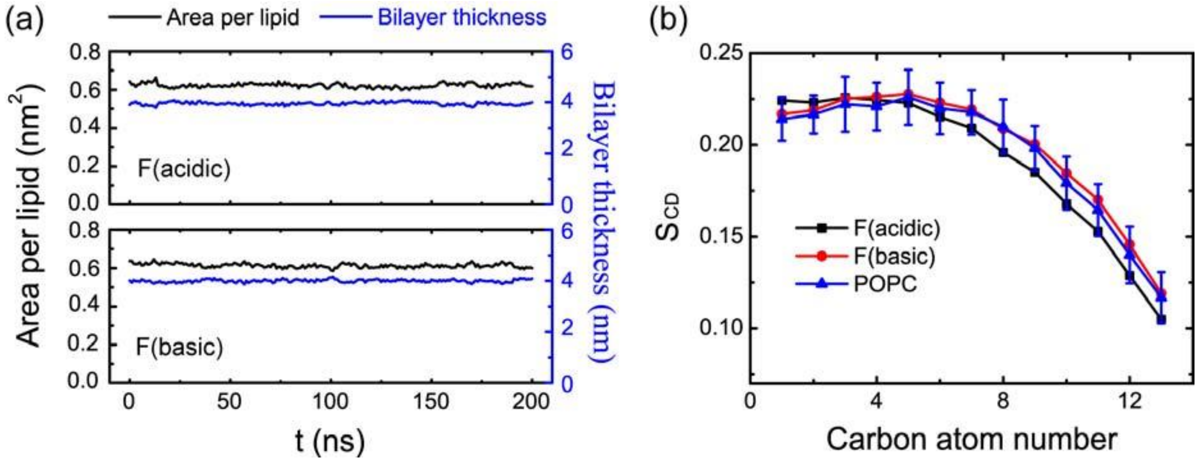

Supplement: Supplementary file 1 [file ijms-14-14532-s001.pdf]
